# Two birds with one stone: An antibiotic hit blocking *Staphylococcus aureus* heme uptake with serendipitous hemoglobin left-shifting activity

**DOI:** 10.1016/j.isci.2026.115625

**Published:** 2026-04-07

**Authors:** Sarah Hijazi, Francesco Marchesani, Marialaura Marchetti, Valeria Buoli Comani, Paul Brear, Barbara Campanini, Luca Ronda, Serena Faggiano, Eleonora Gianquinto, Somayeh Asgharpour Hassankiade, Barbara Rolando, Francesca Spyrakis, Carlotta Compari, Loretta Lazzarato, Omar De Bei, Emanuela Frangipani, Stefano Bettati

**Affiliations:** 1 Department of Biomolecular Sciences, University of Urbino Carlo Bo, Urbino, Marche 61029, Italy; 2 Department of Medicine and Surgery, University of Parma, Parma, Emilia-Romagna 43125, Italy; 3 Department of Food and Drug, University of Parma, Parma, Emilia-Romagna 43124, Italy; 4 Department of Biochemistry, University of Cambridge, Cambridge CB2 1GA, UK; 5 Institute of Biophysics, National Research Council (CNR), Pisa, Tuscany 56124, Italy; 6 Department of Drug Science and Technology, University of Turin, Turin, Piedmont 10124, Italy

**Keywords:** biological sciences, molecular biology, microbiology, structural biology

## Abstract

Infections caused by *Staphylococcus aureus* depend on its ability to access essential nutrients, including acquiring iron from human hemoglobin (Hb) through the iron-regulated surface determinant (Isd) system. The compound 4-[(2-{[5-(1H-indol-3-yl)-1,3,4-oxadiazol-2-yl]sulfanyl}acetyl)amino]benzoic acid (C35) was recently identified as a promising antimicrobial agent for its ability to bind Hb and hamper its interaction with the staphylococcal hemophore IsdB *in vitro*. Here, we show that C35 inhibits *S. aureus* growth by targeting the hemophore-driven iron-acquisition system, highlighting its potential as an inhibitor and validating hemophores as antibacterial targets. Furthermore, for drug design purposes, we solved the X-ray structure of Hb:C35 complex. In contrast to the predicted binding pose, C35 binds tetrameric Hb in a cleft between the α subunits, stabilizing a relaxed conformation (R2) and increasing Hb oxygen affinity. This serendipitous result hints to C35 as a promising scaffold for developing compounds with diverse, or even dual, therapeutic aims, with antimicrobial and Hb-modulating activity.

## Highlights

• Hemophore-mediated iron acquisition in *S. aureus* is inhibited by a small molecule
• C35 binds hemoglobin and blocks bacterial access to heme
• C35 functions as a non-covalent hemoglobin left shifter
• Hemoglobin emerges as a dual target for antimicrobial and Hb-modulating therapies

## Graphical abstract

**Figure:**
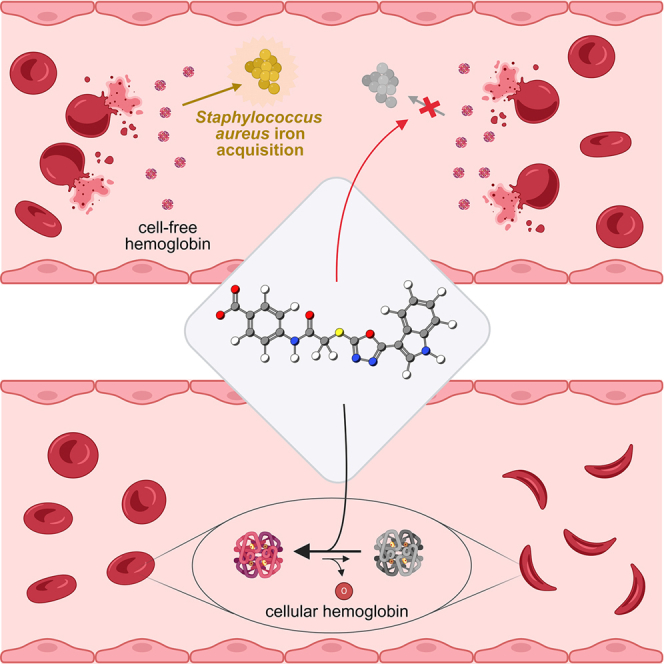

## Introduction

*Staphylococcus aureus* is a human commensal and opportunistic pathogen capable of causing severe infections. Its ability to develop resistance to antibiotics highlights the urgent need for new, effective antimicrobial agents.^1^ *S. aureus* depends on iron for survival; in the human body, this metal is primarily associated with heme cofactor in hemoglobin (Hb). With the aim of acquiring hemic iron, during infection *S. aureus* induces red blood cell (RBC) hemolysis exploiting hemolysins, thus allowing access to free Hb.^2^ Iron gain is mediated by the iron-regulated surface determinant (Isd) system, a nine-protein gear in charge of intercepting Hb, extracting heme, and internalizing it.^3^ During the first step, the cell wall-anchored hemophore IsdB interacts with circulating Hb^4^ and extracts the heme,^5^ which is transferred to other proteins of the Isd system to complete the iron acquisition process.^2^ IsdB is a multifaceted protein that plays a crucial role in *S. aureus* virulence by facilitating iron acquisition, promoting adherence to host tissues, and modulating the host immune response.^6^^,^^7^^,^^8^^,^^9^ Due to its significant role in *S. aureus* pathogenesis, IsdB has been explored as a potential vaccine candidate. However, clinical trials have proven unsuccessful, underscoring the need for innovative antibacterial strategies to combat *S. aureus* infections.^10^ One promising approach is the discovery of chemical entities able to counteract the IsdB:Hb complex formation.^11^ With this purpose, in a recent work, we conducted a virtual screening campaign targeting the IsdB:Hb complex, and *in vitro* binding tests led to the identification of 4-[(2-{[5-(1H-indol-3-yl)-1,3,4-oxadiazol-2-yl]sulfanyl}acetyl)amino]benzoic acid (C35) as the most potent molecule, with a K_D_ for Hb of 0.57 ± 0.06 μM.^12^ These results suggested that C35 could act as a promising antimicrobial agent capable of disrupting iron acquisition in *S. aureus*, thereby leading to bacterial iron starvation and consequent growth inhibition. To test that hypothesis, we now have performed microbiological tests that proved the C35 effectiveness in inhibiting *S. aureus* growth in the presence of Hb as the sole iron source. To provide the basis for chemical optimization of the compound, we also solved the crystallographic structure of Hb in complex with C35.

Unexpectedly, the structural analysis revealed a binding pose different from the one initially hypothesized at the Hb-IsdB interaction surface,^12^ but similar to that of a known class of molecules which allosterically increase the affinity of Hb for oxygen by stabilizing its relaxed, high-affinity state. These molecules are known as left-shifters, as they shift the Hb oxygen-binding curve leftwards, *i.e.*, to lower partial oxygen pressures. From a pharmacological perspective, the biological effect of left-shifters is primarily exploited in the treatment of sickle cell disease (SCD), an inherited disorder in which Glu6 on Hb beta-chains is substituted by a valine.^13^ This mutation promotes Hb polymerization upon deoxygenation that favors the tense, T state. In this context, left-shifters stabilize the relaxed, R state and decrease the amount of T state Hb, hence delaying the polymerization of sickle Hb (HbS).^14^^,^^15^^,^^16^

We found out that C35 actually acts as a strong left-shifter on isolated Hb in solution. After showing the effectiveness of the molecule in inhibiting *S. aureus* growth when Hb is the sole iron source, we thus identified by serendipity a potential hit compound for the development of therapeutic agents for SCD, or even for a dual therapy targeting both SCD and bacterial infections. The latter is a stimulating possibility, given the known susceptibility of SCD patients to infections.^17^

## Results

### C35 inhibits *S. aureus* growth in the presence of Hb as sole iron source

**Figure 1:**
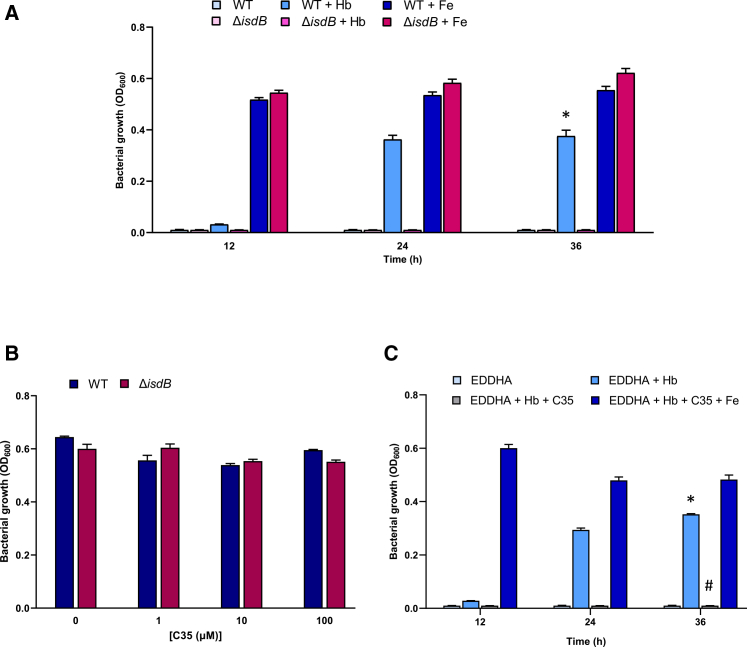
C35 inhibits *S. aureus* growth in the presence of Hb as the sole iron source *S. aureus* WT and its isogenic Δ*isdB* mutant were pre-cultured in RPMI supplemented with 500 μM EDDHA, to restrict iron availability, for 16–20 h at 37°C with good aeration (shaking 180 rpm). The next day, cultures were washed and resuspended in NRPMI supplemented or not with the indicated compounds. (A) Effect of 120 nM Hb (Hb) and 500 μM FeCl_3_ (Fe) on *S. aureus* WT and Δ*isdB* growth, in NRPMI + 500 μM EDDHA. (B) Toxicity of C35 on *S. aureus* WT and Δ*isdB*, grown for 24 h in NRPMI without EDDHA, to allow the growth of both strains. (C) Effect of 100 μM C35 on *S. aureus* WT grown in NRPMI + 500 μM EDDHA (EDDHA) supplemented with 120 nM Hb alone or in combination with 500 μM FeCl_3_. As a negative control, the growth of the WT strain in NRPMI +500 μM EDDHA was also included. Each value is the average of three different cultures ± standard deviation. The symbols indicate statistically significant differences as determined by a Student’s *t* test (*p* < 0.05) in relation to the WT strain grown in the presence of EDDHA with no Hb (∗) or the WT strain grown in the presence of EDDHA with Hb (#).

We have recently identified a compound named C35 that binds Hb with sub-micromolar affinity and interferes with the formation of the IsdB:Hb complex.^12^ To investigate whether C35 could act as antimicrobial agent capable of disrupting iron acquisition in *S. aureus*, thereby leading to bacterial iron starvation and consequent growth inhibition, a mutant lacking the IsdB component of the Isd system was generated in *S. aureus* Newman (*i.e.*, *S. aureus* Δ*isdB*). Since the expression of the Isd system is regulated by the ferric uptake regulator (Fur), which enables its expression under iron-poor conditions, the chemically defined medium NRPMI devoid of iron was used.^18^ To identify the best condition for C35-inhibitory testing, we first investigated the role of Hb in supporting *S. aureus* growth in our experimental setting, using both the WT and the Δ*isdB* strains. Bacteria were pre-cultured in RPMI supplemented with 500 μM 2-[2-[[2-Hydroxy-1-(2-hydroxyphenyl)-2-oxoethyl]amino]ethylamino]-2-(2-hydroxyphenyl)acetic acid (EDDHA) to iron-starve bacterial cells. The following day, cultures were sub-inoculated in NRPMI in the presence of 500 μM EDDHA, supplemented or not with either 120 nM Hb or 500 μM FeCl_3_, and monitored overtime for 36 h (Figure 1A). Results showed that both strains failed to grow in the absence of an iron source, indicating that EDDHA, when added at 500 μM, effectively prevents the acquisition of iron traces present in NRPMI, which would likely occur through the production of siderophores, usually expressed under iron-depleted conditions.^19^ Interestingly, supplementation with 120 nM Hb promoted the growth only of the WT strain but not of the Δ*isdB* (Figure 1A), in line with previous findings.^6^^,^^18^ Notably, this Hb-mediated growth promotion was observed after 24 h post-incubation, suggesting that *S. aureus* requires time to sense the presence of Hb in the medium, and subsequently expresses the IsdB hemophore. In contrast, both strains were able to readily utilize FeCl_3_, showing comparable growth, thus confirming the specificity of IsdB for Hb utilization (Figure 1A). Altogether, these data highlight the phenotypic characterization of the Δ*isdB* mutant, thus providing a foundation for subsequent Hb-inhibitory testing.

C35 toxicity was investigated by monitoring the growth of WT and Δ*isdB* in NRPMI without EDDHA, to allow bacterial growth, in the presence of different C35 concentrations (*i.e.*, 1, 10, and 100 μM), and compared to the one of the positive control, devoid of the compound (Figure 1B). No growth defect was observed for both strains at all C35 concentrations tested, compared to the unamended controls (Figure 1B). Following the toxicity assessment of C35 toward *S. aureus*, the maximum concentration used (100 μM) was then selected to investigate the ability of this compound to inhibit Hb-mediated growth promotion in the WT strain. To this aim, *S. aureus* WT cells were cultivated in NRPMI supplemented with 500 μM EDDHA and Hb, in the presence or absence of C35. Interestingly, C35 completely inhibited the WT growth in the presence of 120 nM Hb as the sole iron source, thus indicating that it is able to block IsdB-mediated Hb uptake in *S. aureus* (Figure 1C), while supplementation with 500 μM FeCl_3_ as an alternative iron source, reversed C35-mediated growth inhibition (Figure 1C). Additional evidence supporting the proposed mechanism of action confirmed that C35 exerts its inhibitory effect by binding to Hb, making it selective for the hemophore-mediated iron acquisition system. Indeed, isothermal titration calorimetry (ITC) measurements excluded any measurable interaction between C35 and IsdB at the tested concentrations (Figure S1).

### Three-dimensional structure of Hb:C35 complex

**Figure 2:**
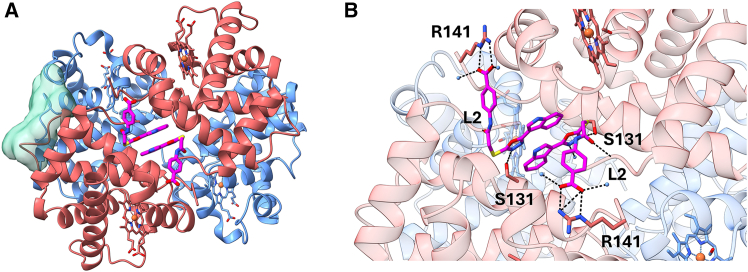
Binding of C35 to Hb (A) The X-ray three-dimensional structure of Hb co-crystallized with compound C35. The α and β subunits are shown as red and blue ribbons, respectively; the semi-transparent green surface represents the putative binding site of C35 as predicted by molecular docking; C35 is shown in magenta. (B) Close-up of the binding site of C35 in the cleft between the two Hb α subunits (in red). β subunits are in blue, C35 in magenta, while contacts are shown as black dashed lines. Water molecules are represented as light blue spheres.

Given the target of C35 to inhibit *S. aureus* growth and aiming to further explore this molecule as a potential hit compound, we investigated the binding of C35 to Hb at atomic resolution. The X-ray crystallographic structure of tetrameric Hb in complex with C35 was obtained by co-crystallization (PDB: 28OD, Figure 2A; Table S1). The final resolution was 1.5 Å and the crystals form showed a symmetry belonging to the space group P 32 2 1.

The binding stoichiometry of C35 to Hb observed in the crystals is 2:1, *i.e.*, two molecules of C35 symmetrically bind one Hb tetramer, confirming the stoichiometry estimated by ITC.^12^ However, the crystallographic structure identified a binding pocket that differs from the one proposed by docking in our recent paper (Figure 2A).^12^ Indeed, the obtained structure shows C35 bound in a cleft between the Hb α subunits. In this pocket, the two C35 molecules form several symmetrical interactions with Hb α lining residues (Figure 2B). Specifically, the benzoic acid moiety of each C35 molecule interacts through a salt bridge with the positively charged lateral chain of Arg141. Each molecule of C35 establishes an H-bond between the oxadiazolic moiety and the side chain of Ser131 and another H-bond between the amide group of C35 and the -NH on the backbone of Leu2. The oxadiazolic and the indolic moieties of both C35 molecules interact with each other through a stacking interaction. Furthermore, being the C35-binding site partially exposed to the solvent, different polar contacts with water molecules are also observed.

### C35 binds Hb in the same cleft of Hb left-shifters and stabilizes an R2 conformation

Structural analysis of the Hb:C35 complex uncovered a previously unreported binding site for C35. To further investigate this new finding, we conducted a detailed comparison of Hb in complex with C35 with the Hb structures deposited in the PDB.

**Figure 3:**
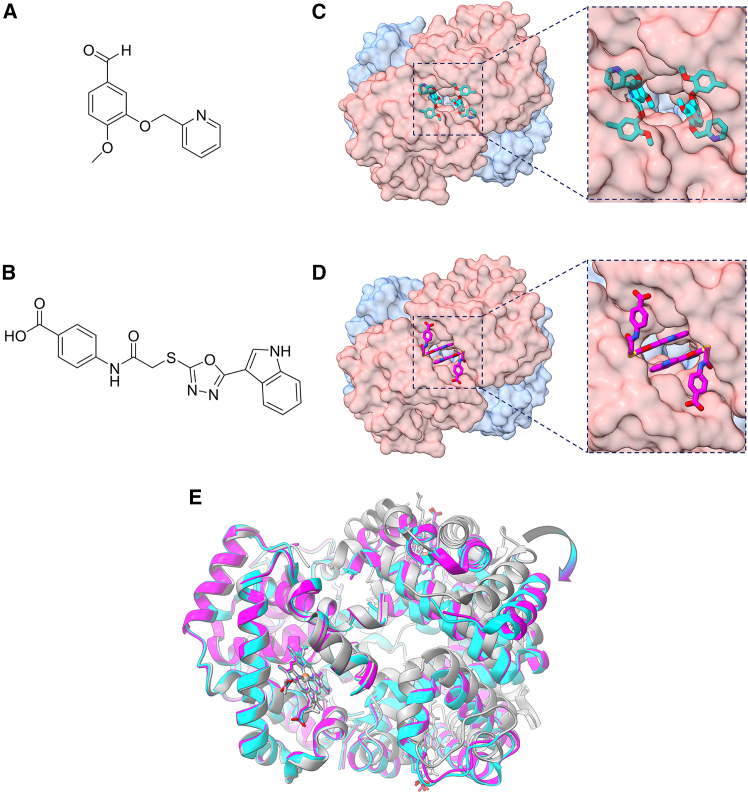
Structural comparison between Hb bound to C35 and Hb bound to INN298 (A) Chemical structure of the compound INN298. (B) Chemical structure of the compound C35. (C) Molecular surface of Hb bound to INN298 and its close-up on the INN298-binding site. (D) Molecular surface of Hb bound to C35 and its close-up on C35 binding site (PDB: 28OD). INN298 and C35 are reported as cyan and magenta sticks, respectively. (E) Different tetrameric Hb structures superimposed on the α1 and β1 chains: HbCO (R state, PDB: 2DN3, gray ribbons), Hb bound to INN298 (R2 state, PDB: 3IC0, cyan ribbons), and Hb bound to C35 (R2 state, PDB: 28OD, magenta ribbons). Heme molecules are represented as sticks. The RMSD values estimated for Hb bound to INN298 or to C35 considering R-state Hb as a reference are 4.46 Å and 4.39 Å, respectively. The RMSD value between INN298- and C35-bound Hbs is 0.54 Å.

This analysis was performed using the ‘‘Structure similarity search’’ tool available on the PDB website (https://www.rcsb.org/search/advanced/structure). The tool performs a comprehensive analysis of the PDB considering the entire protein assembly, including its ternary/quaternary state and any bound molecules. The first ten structures returned by the algorithm are listed in Table S2 and are all human Hb structures in the R conformation. The R state corresponds to a conformation of Hb with high oxygen affinity, where the α1β1 dimer is rotated by 15° with respect to the α2β2 dimer compared to the low oxygen affinity T state. Among the top 5 highest-scoring structures, two presented a ligand bound in a similar position with respect to C35 (*i.e.*, PDB: 1QXE and PDB: 3IC0). In 1QXE, Hb binds a molecule of 5-hydroxymethyl furfural (5-HMF), whereas in 3IC0, it is bound to a vanillin derivative, 4-hydroxy-3-methoxybenzaldehyde (INN298). Both molecules were characterized by Safo’s group as Hb left-shifters,^20^^,^^21^ *i.e.*, ligands that stabilize the R state with respect to the T state, thus increasing the oxygen affinity. These molecules have been developed as potential therapeutic agents against sickle cell anemia. Given its higher potency as an Hb left-shifter compared to 5-HMF, we focused our analysis on the comparison between C35 and INN298 (Figures 3A and 3B). INN298 and C35 share the same binding site on Hb (Figures 3C and 3D); however, they markedly differ in binding geometry and stoichiometry (Figure S2). While C35 binds the Hb tetramer non-covalently with a 2:1 stoichiometry (*vide supra*), INN298 occupies multiple adjacent subpockets and achieves affinity through a more distributed interaction pattern, associating with Hb at a 4:1 stoichiometry per tetramer. In this case, two molecules form a covalent bond with the N-terminal Val1 of the α chains, whereas the remaining two interact non-covalently.

An additional informative structural comparison is provided by PF-07059013 (PDB:7JY3), a non-covalent Hb left-shifter developed for the treatment of SCD and reported to bind the same interfacial pocket.^22^ Owing to its non-covalent-binding mode, comparison of PF-07059013 with C35 is limited here to spatial features, revealing a compact and centrally confined binding geometry dominated by localized H-bond and solvent-mediated interactions (Figure S2). Although the three Hb structures bound to C35, INN298, and PF-07059013 are overall similar, the closest quaternary correspondence is observed between the C35- and INN298-bound complexes. For this reason, subsequent analyses focused on the comparison between C35 and INN298.

It is well known that compounds able to interact with Hb in the allosteric-binding site between the α subunits can stabilize a relaxed R conformation. In particular, INN298 stabilizes an alternative quaternary Hb conformation named R2 state, over the R state trajectory.^23^^,^^24^ The R and R2 states of tetrameric Hb mainly differ in the positioning of the α2β2 dimer relative to the α1β1 dimer. Therefore, to investigate the effect of C35 on Hb quaternary structure, we compared C35-bound Hb with reference structures by calculating the global root-mean-square deviation (RMSD) on the α2β2 subunits after superimposing the α1β1 subunits. As references, we used R-state HbCO (PDB: 2DN3,^25^) and R2-state Hb bound to INN298 (PDB: 3IC0) (Figure 3E). The RMSD calculated relative to the R2 structure stabilized by INN298 is very low (0.54 Å) compared to that obtained relative to the R structure (4.39 Å). Taken together, these results suggest that C35, similarly to INN298, can stabilize an R2 Hb conformation and thus acts as an Hb allosteric modulator.

### Compound C35 increases Hb oxygen affinity

Considering that C35 and the left-shifter INN298 share the same binding site and induce comparable changes in Hb structure, we decided to compare the effect of the two molecules on the functional properties of Hb, *i.e.*, on its oxygen-binding affinity and cooperativity.

INN298, stabilizing the R2 conformation, reduces the binding cooperativity of oxygen to Hb and increases the oxygen affinity, thus also slowing down the HbS polymerization process that involves the deoxy form of the protein.^14^^,^^21^ However, in the reference study,^21^ the effect of INN298 on oxygen binding was only investigated in intact RBCs, thus, in addition to its direct action on Hb, the permeation of the effector into the cells must also be considered. Here, we decided to investigate the activity of INN298 at the highest concentration tested by Abdulmalik et al. (*i.e.*, 2 mM).^21^

**Figure 4:**
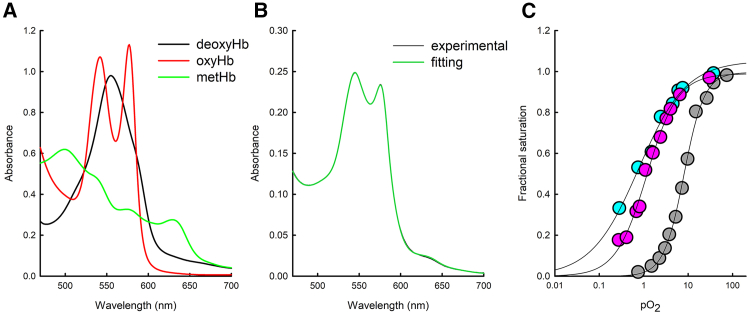
Effect of C35 and INN298 on the oxygen binding to Hb (A) Reference spectra used for linear combination: deoxyhemoglobin (deoxyHb), oxyhemoglobin (oxyHb), and methemoglobin (metHb). (B) Example of comparison between an experimental spectrum (black line) measured at a pO_2_ of 9.4 torr and the corresponding fit to the linear combination of reference spectra (green line). (C) Oxygen-binding curves of Hb measured at 37°C in the absence (gray circles) and presence of either 1 mM C35 (magenta circles) or 2 mM INN298 (cyan circles). The data points represent the Hb fractional saturation with oxygen obtained from the linear combination of reference spectra. Lines through data points are the fitting to the Hill equation (Equation 3). The estimated values are reported in Table 1.

The reference oxygen-binding curve of Hb showed a *p*50 (*i.e.*, the pO_2_ corresponding to 50% fractional saturation) of 8.11 ± 0.29 torr, with a Hill coefficient (n) of 1.90 ± 0.10 (Figure 4; Table 1). Stripped Hb (*i.e.*, Hb outside RBCs in the absence of any allosteric effector) has a reported n of approximately 2.4.^26^ The lower value measured here is likely due to the effect of DMSO (used at a concentration of 2% (v/v) to align with studies conducted with INN298 and C35), as reported in the work of Liu and colleagues.^27^ As expected, INN298 shifted to the left in the oxygen-binding curve of Hb, with a *p*50 value of 0.77 ± 0.11 torr in the presence of the compound (Figure 4; Table 1). Also, cooperativity was found to be strongly affected by the presence of INN298, with a Hill coefficient dropping to 0.81 ± 0.10.

**Table 1:** Hb oxygen-binding parameters (*p*50 and Hill coefficient) of Hb in the absence and presence of either 2 mM INN298 or 1 mM C35 at pH 7.4, 37°C

	Reference	+2 mM INN298	+1 mM C35
p50 (torr)	8.11 ± 0.29	0.77 ± 0.11	1.17 ± 0.09
n (Hill)	1.90 ± 0.10	0.81 ± 0.10	1.25 ± 0.09

The oxygen-binding curve in the presence of C35 was measured at a final concentration of 1 mM compound. This concentration is significantly higher than its dissociation constant (K_D_ = 0.57 ± 0.06 μM^12^) but was necessary to saturate Hb, which was present at a concentration of 100 μM in the analysis to allow monitoring of the absorption of Q bands for the calculation of the fractional saturation (Figures 4A and 4B). The p50 calculated in the presence of C35 was 1.17 ± 0.09 torr (Figures 4C; Table 1), comparable to the value obtained in the presence of INN298. Also in this case, the cooperativity of oxygen binding is almost completely lost, with a Hill coefficient of about 1.25 ± 0.09 (Figures 4C; Table 1).

### C35 does not impair haptoglobin binding to Hb

**Figure 5:**
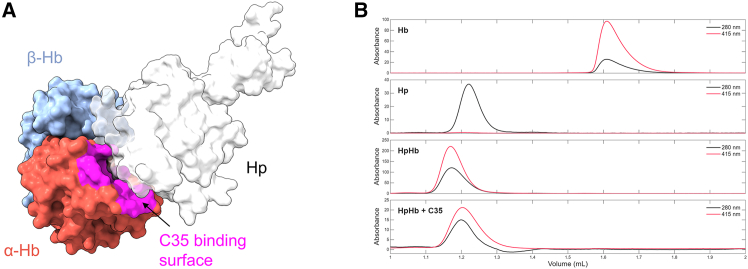
Effect of C35 binding on the formation of the Hb:Hp complex (A) Surface representation of an Hb dimer derived from the crystal structure reported in this study (PDB ID: 28OD). The Hb α-chains are shown in red and the Hb β-chains in blue. The region of the Hb surface contacted by C35 in a Hb tetramer as from the crystal structure is highlighted in magenta. For comparison, a single haptoglobin (Hp) protomer from the Hb:Hp complex (PDB: 4F4O) was structurally aligned onto the Hb dimer; the surface of Hp is displayed as a semi-transparent white surface. (B) SEC chromatograms of isolated Hb, isolated Hp, and the Hb:Hp complex in the absence and presence of 1 mM C35. Protein and heme signals were monitored at 280 nm (black line) and 415 nm (red line), respectively.

In the crystal structure of tetrameric Hb with C35, the compound binds at the interface between the two Hb dimers, specifically at the level of the α subunits. Under physiological conditions, haptoglobin (Hp) binds free Hb dimers in the bloodstream via specific non-covalent interactions, forming a stable complex that prevents renal loss and oxidative damage by enabling Hb clearance through CD163-expressing macrophages.^28^ During the initial virtual screening campaign, C35 was selected to interact with a region of Hb that was predicted not to interfere with Hp binding.^12^ However, the binding pose revealed by the crystal structure differs from the initially predicted one. In this experimentally observed configuration, C35 binds at a site that is proximal to the Hb:Hp interaction interface. Structural superposition of a single Hb dimer extracted from the tetrameric Hb:C35 crystal structure with the crystal structure of the Hb:Hp complex reveals that the C35-binding site partially overlaps with a region of Hb involved in Hp recognition (Figure 5A). Although this observation does not directly imply functional interference, it raised the possibility that C35 binding could be incompatible with Hp association.

This structural insight prompted us to experimentally investigate whether C35 affects Hb:Hp complex formation, as such interference would represent a serious limitation for the development of C35 as an antibacterial agent acting in the extracellular plasma compartment.

To explore the effect of C35 on Hb:Hp interaction, we performed size-exclusion chromatography (SEC) experiments. As a first step, experimental conditions were optimized to isolate the Hb:Hp complex chromatographically. Hb and Hp were mixed in solution at a 2:1 stoichiometric ratio (one Hb dimer per Hp protomer), and the resulting chromatogram was compared to those of the individual proteins run separately (Figure 5B). The chromatogram of the Hb-Hp mixture showed a single peak with a lower elution volume (1.19 mL) than each of the separate proteins (1.22 mL for Hp and 1.61 mL for Hb), indicating the formation of a higher molecular weight complex. Given that the apparent molecular size of Hp in SEC appeared to be significantly influenced by its extensive glycosylation, which causes Hp to elute at lower volumes than expected from its molecular weight, the contribution of Hb to the overall chromatographic profile of the complex results in only a modest additional shift in the elution volume. Accordingly, no residual peaks corresponding to the individual proteins were observed for the 2:1 Hb:Hp sample, indicating that the complex was successfully isolated under these conditions. To provide more direct evidence for complex formation, chromatograms were monitored at both 280 nm, reporting on total protein content, and 415 nm, which specifically tracks the presence of heme and thus of Hb. Notably, the heme signal co-eluted exclusively with the Hp-containing peak, and no free Hb/heme peak was detected at higher elution volumes, confirming a stable Hb:Hp complex formation (Figure 5B).

Maintaining the same protein concentrations and stoichiometry, the SEC experiment was repeated in the presence of C35, added at a concentration of 1 mM to both the sample and the mobile phase. While the resulting chromatographic peak appeared slightly broader compared to the control, the elution volume remained unchanged and the heme signal continued to co-elute with the Hp-containing peak, with no evidence of unbound Hb. These observations indicate that C35 does not disrupt the Hb:Hp complex under the tested conditions, suggesting that Hp binding dominates over C35 association because C35 binding is not sufficiently strong to interfere with Hb:Hp complex formation. Consequently, no off-target effects related to Hb:Hp dissociation are expected to be associated with the administration of this compound.

## Discussion

The Isd system plays a central role in iron acquisition and is a key contributor to *S. aureus* pathogenicity, as demonstrated in several murine models of infection.^6^^,^^29^^,^^30^^,^^31^ Among its components, IsdB is particularly critical: *isdB* deletion mutants consistently exhibit attenuated virulence, including significantly reduced bacterial burdens in systemic infection models and diminished kidney abscess formation.^6^^,^^30^^,^^32^ These findings underscore the essential role of IsdB in heme acquisition from Hb and pathogenesis, identifying it as a promising target for the development of anti-*Staphylococcus* strategies.^3^^,^^6^^,^^31^ Disrupting the IsdB:Hb interaction could indeed represent an effective approach to inhibit *S. aureus* growth and prevent infection. In this work, we report the unexpected identification of a dual activity for the small molecule C35, which functions as an allosteric effector of Hb that enhances oxygen affinity and as a potent antimicrobial agent inhibiting *S. aureus* growth through interference with iron acquisition. Originally selected for its ability to disrupt the IsdB:Hb interaction *in vitro*, C35 was shown to effectively inhibit *S. aureus* growth under iron-limited conditions, an effect that was reversed by excess of FeCl_3_, strongly suggesting iron starvation as the underlying antibacterial mechanism.

Structural studies revealed that C35 binds in a solvent-exposed cleft between the α subunits of the Hb tetramer, a site shared with known Hb left-shifters such as INN298^21^ and Voxelotor.^33^ This binding mode, distinct from the originally predicted docking pose, stabilizes an R2 quaternary conformation of Hb, characterized by high oxygen affinity and reduced cooperativity. Unlike most known left-shifters, which form covalent adducts with Hb, C35 interacts via non-covalent forces, representing a prototype molecule for a distinct class of Hb modulators. Notably, this binding mode is, to date, shared only by PF-07059013,^22^ a non-covalent Hb left-shifter that recently entered clinical phase 1 trials and exhibits a similar interaction interface with nanomolar affinity for Hb (https://clinicaltrials.gov/study/NCT04323124). This convergence further underscores the growing pharmacological interest in non-covalent Hb modulators as safer and potentially more tunable therapeutic alternatives for the treatment of SCD.

The inhibition of *S. aureus* growth in the presence of C35 raised the question of whether this activity could be linked to altered Hb oligomerization dynamics. Specifically, since heme extraction by the Isd system requires Hb dimerization,^34^ it was hypothesized that C35 may exert its inhibitory effect by stabilizing the tetrameric form of Hb, thereby limiting the accessibility of heme to IsdB. However, experimental analysis by SEC demonstrated that C35 does not significantly affect the tetramer-to-dimer equilibrium of Hb (Figure S3), suggesting that the primary mechanism of action is not related to oligomeric stabilization but rather to direct competition with hemophore binding.

It should be noted, however, that the binding mode identified by X-ray crystallography reflects the interaction of C35 with tetrameric Hb, which is favored under the high protein concentrations required for crystal formation, whereas Hb is expected to be mostly in the dimeric state at the concentrations used for the initial screening^12^ and the *S. aureus* growth inhibition assay. Under these conditions, C35 may therefore engage a site on dimeric Hb that partially or fully overlaps with the IsdB-binding interface, thereby providing a plausible explanation for the observed inhibition of bacterial growth. The high Hb concentration required for crystallization shifts the dimer-tetramer equilibrium toward the tetramer, hampering further investigation of the binding mode by crystallographic techniques.

The presented data demonstrate that a single ligand can simultaneously modulate oxygen binding to Hb, through the stabilization of a specific Hb quaternary structure, and interfere with heme scavenging by bacterial hemophores. This discovery opens new avenues for the development of dual-acting compounds that target both the functional modulation of Hb and bacterial iron acquisition. In terms of antimicrobial activity, moreover, it is worth noting that several bacterial iron acquisition systems rely on heme extraction from Hb.^35^^,^^36^ Given that C35 exerts its effect through direct binding to Hb, it is conceivable that it could interfere with other bacterial iron acquisition systems that similarly exploit heme scavenging from host sources.

The clinical relevance of such dual-acting agents is underscored by the increased susceptibility of SCD patients to bacterial infections.^17^ For this population, bifunctional C35 derivatives with balanced intra- and extracellular distribution could simultaneously address both pathological Hb polymerization and the associated infectious risk. Although the concentrations required to modulate intracellular and extracellular targets may differ substantially, it can be speculated that compounds favoring RBC uptake might still retain sufficient plasma levels to exert antibacterial activity, thereby offering a dual therapeutic benefit even with a predominant intracellular partitioning. At the current stage, C35 should be regarded as a proof-of-concept scaffold rather than a clinically viable compound, and its pharmacokinetic and distribution properties—particularly RBC permeability—will require careful optimization before any therapeutic application can be envisaged.

In conclusion, although further *in vivo* studies are warranted to evaluate the antibacterial efficacy of C35 in murine infection models, our study highlights the therapeutic potential of targeting the α subunit interface of Hb as a previously unexplored pharmacological hotspot, offering a promising platform for the development of compounds that combine hemoglobinopathy treatment with antimicrobial action.

### Limitations of the study

The study provides evidence of inhibition of *S. aureus* growth by a small molecule hampering the IsdB:Hb interaction and mechanistic insight into the unexpected allosteric modulation of Hb. However, some aspects remain to be clarified. The antibacterial effect has not yet been validated in an *in vivo* model, where *S. aureus* could exploit alternative siderophore-mediated iron acquisition pathways. Although the crystal structure of the molecule in complex with Hb elucidates the structural basis for its ability to enhance oxygen affinity, the precise mechanism of bacterial growth inhibition is not fully resolved. In particular, it remains unclear whether the inhibitory effect arises from binding at the same site or at an alternative site on Hb, which is likely to exist mostly as a dimer under conditions relevant for bacterial iron acquisition.

Additionally, the low concentrations at which the molecule exhibits activity *in vitro* are not directly translatable to crystallographic assays, limiting structural observation of the inhibitory interaction. Another promising but unexplored facet concerns whether the molecule can interfere with heme-dependent iron acquisition in other pathogens, potentially broadening its therapeutic applicability.

## Resource availability

### Lead contact

Requests for further information and resources should be directed to and will be fulfilled by the lead contact, Omar De Bei (omar.debei@unipr.it).

### Materials availability

This study did not generate new unique reagents. The C35 compound is commercially available.

### Data and code availability

The dataset generated and analyzed in this study has been deposited in the Figshare Database: https://doi.org/10.6084/m9.figshare.29145080. The crystallographic model presented in this study is available under accession code PDB:28OD.

## Acknowledgments

This project was funded by “PRIN-2020— Defeat antimicrobial resistance through iron starvation in *Staphylococcus aureus* (ERASE)” (grant 2020AE3LTA) to F.S., E.F., and S.B.; PRIN 2022RCP52Y “BIOMHEME - A biomimetic approach to remove free hemoglobin from plasma inspired by bacterial iron capturing systems” Italian Ministry of University and Research to L.R.; Project funded by the 10.13039/501100000780European Union – Next Generation EU, Mission 4 Component 1, CUP D53D23010790006. This work has been carried out in the frame of the ALIFAR project of the University of Parma, funded by the Italian Ministry of University through the program ‘Dipartimenti di Eccellenza 2023–2027’. We thank the 10.13039/501100006692University of Turin for funding support (SPYF_RILO_23_01; GIAE_RILO_24_02; LAZL_RILO_23_01). We thank Maisem Laabei, University of Bristol, UK, for kindly providing *Staphylococcus aureus* Newman, *Escherichia coli* IM08B, and the pIMAY∗ plasmid. We thank Gianmarco Mangiaterra (University of Urbino Carlo Bo, Urbino, Italy) for technical assistance in constructing the pIMAY∗Δ*isdB* plasmid. The authors would like to thank Diamond Light Source for beamtime (proposal mx25402), and the staff of beamlines I03 for assistance with crystal testing and data collection. We thank Dr. Davide Cavazzini (Department of Chemistry, Life Sciences and Environmental Sustainability, University of Parma) for technical support in size-exclusion chromatography experiments. This work is dedicated to the memory of Prof. Andrea Mozzarelli, full professor of Biochemistry at the University of Parma who sadly passed away on August 17^th^, 2024. We are most grateful to Prof. Mozzarelli for his seminal work on hemoglobin allosteric regulation and kinetic mechanisms of sickling that inspired many of us and a plethora of students throughout his scientific and academic career.

## Author contributions

S.B., E.F., L.R., O.D.B., M.M., F.S., and F.M. designed the project and conceptualized the approach; S.B., E.F., B.C., L.R., F.S., L.L., and S.F. supervised the project; S.B., F.S., and E.F. provided financial resources; E.G., S.A.H., B.R., F.S., and L.L. designed, synthesized, and purified C35 compound for assays; S.H. performed the microbiological assays and analyzed data; F.M., M.M., V.B.C., and O.D.B. performed the biochemical characterization and analyzed data; C.C., V.B.C., and S.F. performed the ITC measurements and analyzed data; P.B. performed protein crystallization and structural analysis; S.H., F.M., M.M., V.B.C., E.G., S.A.H., C.C., and O.D.B. prepared the figures; F.M., S.B., B.C., and O.D.B. wrote the original draft; all authors have read and agreed to the published version of the manuscript.

## Declaration of interests

The authors declare no competing interests.

## STAR★Methods

### Key resources table

**Table:** 

REAGENT or RESOURCE	SOURCE	IDENTIFIER
**Bacterial strains**		

*Staphylococcus aureus* strain Newman	Baba et al., 2008	Newman strain
*Staphylococcus aureus* Δ*isdB* mutant	This study	N/A
*Escherichia coli* BL21	Novagen	N/A
*Escherichia coli* IM08B	Monk et al., 2015	N/A

**Biological samples**		

Human red blood cells	Local blood transfusion center	Italian law 219/2005 compliant
Human hemoglobin (Hb)	This study	purified from human RBCs

**Chemicals, peptides, and recombinant proteins**		

C35	This study/Enamine Ltd.	Product code Z54346381
Dimethyl sulfoxide (DMSO)	Merck (Sigma-Aldrich)	Cat# D2650
Potassium hydroxide	Supelco	Cat# 1.05033.1000
Carbon disulfide	Sigma Aldrich	Cat# 180173-500ML
EtOH	VWR	Cat# 20821.330
Potassium carbonate	Alfa Aesar	Cat# A16625
Acetone	Sigma Aldrich	Cat# 24201-2.5L
Tryptic Soy Agar	Liofilchem, S.r.l.	Cat# 610052, 500 G
Tryptic Soy Broth	Liofilchem, S.r.l.	Cat# 610053, 500 G
Iron(III) chloride hexahydrate	Sigma-Aldrich	Cat# 31232-250G-M
Glycerol	Sigma-Aldrich	Cat# G7893-500 mL
Chelex® 100 Resin	Bio-Rad Laboratories	Cat# 142–2822, 500 G
RPMI-1640 medium	Sigma-Aldrich	Cat# R8755-10X 1L
Bacto^TM^ Casamino acids	Gibco, Thermo-Fisher Scientific	Cat# 223050
2-[2-[[2-Hydroxy-1-(2-hydroxyphenyl)-2-oxoethyl]amino]ethylamino]-2-(2-hydroxyphenyl)acetic acid (EDDHA)	BLDpharmatech GmbH	Cat# BD231901-1G
Zinc chloride	Sigma-Aldrich	Cat# 96468-50 G
Manganese(II) chloride	Sigma-Aldrich	Cat# 805930
Calcium chloride dihydrate	Sigma-Aldrich	Cat# C3881-500G
Magnesium chloride anhydrous	BDH Limited Poole England	Cat# 26123
Hydrochloric acid 37%	PanReac AppliChem	Cat# 131020.1212
Water for Injection (saline)	Eurospital	Cat# 40–613/F
Sodium phosphate dibasic	Sigma-Aldrich	Cat# 71640-1KG
Sodium phosphate monobasic	Sigma-Aldrich	Cat# 71496-1KG
Yeast extract	PanReac AppliChem	Cat# A1552,1000
Tryptone	PanReac AppliChem	Cat# A1553,1000
Sodium chloride	PanReac AppliChem	Cat# 131659.1211
Phenylmethanesulphonyl fluoride (PMSF)	Apollo Scientific	Cat# PC6222M
Benzamidine	Fluka	Cat# 12072
Pepstatin A	PanReac AppliChem	Cat# A2205,0010
Lysozyme from chicken egg white	Sigma-Aldrich	Cat# 62971-10G-F
Ethylenediaminetetraacetic acid (EDTA) disodium salt 2-hydrate	PanReac AppliChem	Cat# 131669.1210
HEPES	PanReac AppliChem	Cat# A1069,0500
Sodium hydroxide	PanReac AppliChem	Cat# 141929.1211
Agar	PanReac AppliChem	Cat# A3477,0500
Ampicillin sodium salt	Sigma-Aldrich	Cat# A9518
Anhydrotetracycline	Cayman Chemical	Cat# 10009542
Strep-tag® II-IsdB (residues 125–485)	This study	UniProt Q8NX66
Hayashi reducing system	Hayashi et al.	https://doi.org/10.1016/0005-2795(73)90110-4
Helium 99.999% (5.0)	Nippon gases	N/A
Oxygen 99.5% (2.5)	Nippon gases	N/A
Tris	Merck (Sigma-Aldrich)	T6066-1KG
Gel Filtration HMW Calibration Kit	Cytiva	Cat# 28403842
Haptoglobin (phenotype 1-1)	Athens Research & Technology	Cat# 16-16-080116-1/1
Ammonium sulfate	Merck (Sigma-Aldrich)	Cat# A4418
2-Methyl-2,4-pentanediol (MPD)	Merck (Sigma-Aldrich)	Cat# 332615
4-Chloro-DL-phenylalanine (PCPA)	Sigma-Aldrich	Cat# C6506-5 G
FastDigest restriction enzyme_PstI	Thermo Fisher Scientific	Cat# FD0614
FastDigest restriction enzyme_HindIII	Thermo Fisher Scientific	Cat# FD0504
FastDigest restriction enzyme_XhoI	Thermo Fisher Scientific	Cat# FD0694

**Deposited data**		

C35-bound human hemoglobin structure	Protein DataBank	PDB: 28OD

**Oligonucleotides**		

UPFWisdB	This study	5′-AACTGCAGCCAAAC CGTGTTAAACAATG-3′
UPRVisdB	This study	5′-CCCAAGCTTGTTCAT GTTGTAGAAACAAC-3′
DWFWisdB	This study	5′-CCCAAGCTTAACTAAT AAATCGTCTTTATATTT-3′
DWRVisdB	This study	5′-CCGCTCGAGTGCTA GATTCACAAACGG-3′
OUTFWisdB	This study	5′-TGTATACATAGGCGCAGACA-3′
OUTRVisdB	This study	5′-AACTCGCGGTCTATTGCCA-3′
T3	T3 promoter	5′-AATTAACCCTCACTAAAGGG-3′
T7	T7 promoter	5′-GTAATACGACTCACTATAGGGC-3′

**Recombinant DNA**		

pIMAY∗	Schuster et al.,2019	temperature-sensitive shuttle vector- counter selectable marker PheS∗
pIMAY∗Δ*isdB*	This study	N/A
pASK-IBA3-plus:Strep-tag® II-IsdB	Gianquinto et al., 2019	Vector for the expression of IsdB construct (residues 125–485, UniProt Q8NX66)

**Software and algorithms**		

CCP4 Software Suite (v9.0.0.11)	CCP4 Consortium	RRID:SCR_007255
Phaser (v9.0.0.11)	CCP4 Consortium	RRID:SCR_014219
Coot (v0.9.8.95)	MRC Laboratory of Molecular Biology	RRID:SCR_014222
Grade (v1.2.19)	Global Phasing Ltd.	https://www.globalphasing.com/buster/manual/grade/manual/index.html
Buster (v23.01.2024)	Global Phasing Ltd	https://www.globalphasing.com/buster/
MicroCal PEAQ-ITC Analysis Software (v1.41)	Malvern Panalytical	RRID:SCR_023795
SigmaPlot 12.0	Grafiti LLC	RRID:SCR_003210; http://www.sigmaplot.com/products/sigmaplot/
ChimeraX (V1.10.1)	UCSF Resource for Biocomputing, Visualization, and Informatics	RRID:SCR_015872
MATLAB R2025a	The MathWorks Inc.	https://it.mathworks.com/products/matlab.html
GraphPad Prism (v8.0.1)	GraphPad	RRID:SCR_002798; http://www.graphpad.com/

**Other**		

SPARK® 10M Multimode Plate Reader	Tecan	SPARK 10M
MicroCal PEAQ-ITC	Malvern Panalytical	N/A
MicroPulserTM	Bio-Rad	Cat# 165-2100
Cary 4000 UV–Vis Spectrophotometer	Agilent Technologies	N/A
ÄKTA Pure 25 M	GE Health Sciences™, Chicago, IL, USA	N/A
ÄKTA Prime Plus	GE Health Sciences™, Chicago, IL, USA	N/A
Superdex® 200 Increase 3.2/300	GE Health Sciences™, Chicago, IL, USA	Cat# 29036232
Superdex® 75 Increase 5/150 GL	GE Health Sciences™, Chicago, IL, USA	Cat# 29148722
Diamond Light Source beamline I03	Diamond Light Source (UK)	I03
Corning® filter system	Corning	431097, 0.22 μm PES
CM Sephadex™ C-50 ion exchange resin	Cytiva	Cat# 17022002
Agilent 1260 HPLC system	Agilent Technologies, Inc., Santa Clara, CA, USA	N/A
Environics 4000 gas mixer	Environics Inc, Tolland, CT, U.S.A.	N/A

### Experimental model

Bacterial strains and plasmids used in this study are listed in the key resources table. Bacteria were routinely grown in Tryptic Soy Broth (TSB) with good aeration (shaking at 180 rpm) or in TSB-Agar.

When required, media were supplemented with 10 μg/mL chloramphenicol. Bacteria strains were maintained as frozen stock at −80°C in 20% glycerol.

### Method details

#### Chemicals and solubilization of C35

All reagents were purchased by MERCK (St. Louis, MO, USA) and were used as received. C35 was synthesized in-house, with a purity of at least 95%, as reported in Cozzi et al.^12^ (see Scheme) and dissolved in 100% dimethyl sulfoxide (DMSO, Sigma-Aldrich) at a final concentration of 100 mM. It is also commercially available (Product code Z54346381, Enamine Ltd. https://enaminestore.com/).

**Figure:**
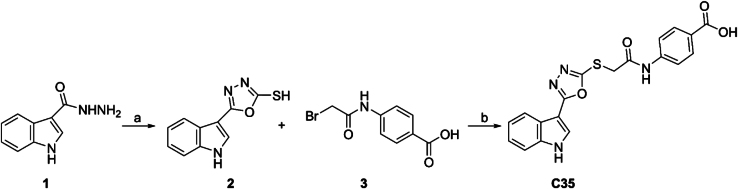

a)KOH, CS_2_, EtOH, reflux, 4 h. b) K_2_CO_3_, acetone reflux.

#### Bacterial strains and growth conditions

All experiments were carried out with *S. aureus* strain Newman^37^ and its isogenic in frame-deletion mutant strain (Δ*isdB*). All media and solutions were prepared with deionized, double distilled water (ddH_2_O). In this study, two media were used: *i)* Roswell Park Memorial Institute (RPMI-1640, Sigma-Aldrich) broth prepared according to the manufacturer’s instructions with a modification; RPMI was supplemented with 1% casamino acids (CAA) (w/v) to support bacterial growth, and *ii)* metal-depleted RPMI (NRPMI) prepared following the protocol described by Pishchany et al.^18^ Briefly, the RPMI medium was treated for 16 h at 4°C with 70 g/L of the metal-chelating Chelex resin (Bio-Rad) under moderate stirring and then filtered through Whatman no. 1 filter paper. The medium was subsequently supplemented with non-iron metals: 25 μM ZnCl_2_, 25 μM MnCl_2_, 100 μM CaCl_2_, and 1 mM MgCl_2_ prepared in advance as sterile 1,000× solutions. The pH of the NRPMI was then adjusted to 7.4, sterilized by filtration using Corning filter system (Dow Corning), and stored at 4°C.

When required, 2-[2-[[2-Hydroxy-1-(2-hydroxyphenyl)-2-oxoethyl]amino]ethylamino]-2-(2-hydroxyphenyl)acetic acid (EDDHA) was resuspended in anhydrous ethanol to a final concentration of 100 mM for sterilization and then added to the culture medium at the required concentration. FeCl_3_ (Sigma-Aldrich) was prepared as a 0.1 M stock solution in 10 mM HCl and stored at −20°C. Human hemoglobin (Hb) was purified from red blood cells (see below) and used to prepare a 930 μM stock solution (on chain basis), which was stored at −80°C in phosphate-buffered saline (PBS). For C35, a 100 mM stock solution was freshly prepared in DMSO and stored at −20°C.

The ability of *S. aureus* WT and its isogenic Δ*isdB* to grow in NRPMI was investigated by monitoring bacterial growth (OD_600_) over time, as described in Pishchany et al.^18^ For that, *S. aureus* WT and its isogenic Δ*isdB* mutant were pre-cultured in RPMI supplemented with 500 μM EDDHA, to restrict iron availability, for 16–20 h at 37°C with 180 rpm shaking. The next day, cultures were centrifuged for 5 min at 7,500 *x g* and the pellets were resuspended in NRPMI containing 500 μM EDDHA. Bacterial suspensions were then subcultured to obtain a final OD_600_ = 0.003 in 200 μL of NRPMI, supplemented with either 500 μM EDDHA and/or 120 nM Hb and/or 100 μM C35, using 96 well-microtiter plates. For the C35 toxicity assay, bacterial cultures from ON were washed and subcultured in 200 μL of NRPMI without EDDHA in the presence of increasing concentrations (0–100 μM). Plates were incubated at 37°C, and bacterial growth (OD_600_) was measured in a multiplate reader (SPARK 10M TECAN) for up to 36 h.

#### Construction of plasmids for molecular cloning

Oligonucleotides used in this study are listed in the key resources table. *S. aureus* ΔisdB was obtained following the mutagenesis protocol described in Schuster et al.^38^ For inactivation of the *isdB* gene, a 955-bp fragment overlapping the ATG of *isdB* and a 989-bp fragment overlapping the TAA of *isdB* were amplified by PCR using the primer couples UPFW*isdB*/DWFW*isdB* and UPRV*isdB*/DWRV*isdB*, respectively, The PCR-amplified upstream and downstream regions of each gene were digested with PstI-HindIII and HindIII-XhoI, respectively, and cloned PstI-XhoI into pIMAY∗, yielding pIMAY∗Δ*isdB*. Then, the latter was introduced by electroporation into *S. aureus* wild type. To enable high-efficiency plasmid transfer in *S. aureus* Newman, cloning procedures were performed in *Escherichia coli* IM08B, a strain that mimics the methylation profiles of major *S. aureus* lineages. The functionality of the plasmid was examined using a temperature-sensitivity test, confirming the ratio of colonies on plates incubated at 28°C and 37°C. Chromosomal integration via a single crossover event was achieved by incubating *S. aureus*/pIMAY∗Δ*isdB* transformants at 37°C with antibiotic selection and verifying by a colony PCR using the T3/T7 primers. The second crossing-over event was encouraged by culturing/sub-culturing the selected clones at 28°C for at least 50 generations, followed by verification of plasmid loss using *para*-chlorophenylalanine (PCPA), a toxic phenylalanine analog. Once chloramphenicol sensitivity was confirmed, genomic DNA was extracted from potential candidates and the deletion was confirmed by PCR using primers OUTFW*isdB*/OUTRV*isdB*, and PCR fragments were checked by sequencing.

#### Protein purification

Human Hb was purified from outdated blood donated by non-smoking volunteers to a blood transfusion center, following the procedure described in Viappiani et al.^38^ Written informed consent was obtained from all donors, and both the blood donation and the use of outdated samples complied with Italian law 219/2005 on blood donation and usage. RBCs were washed with saline and lysed under hypotonic conditions by adding seven volumes of Buffer Hb1 (10 mM HEPES pH 6.9, 1 mM EDTA). The lysate was clarified by centrifugation (23,000 × g, 1 h, 4°C), and the supernatant containing oxygenated Hb was dialyzed against Buffer Hb1. The sample was then loaded onto a CM-Sephadex C-50 column (100 × 5 cm). Soluble RBC components were separated from Hb using a linear gradient from 0% to 80% Buffer Hb2 (10 mM HEPES pH 8.6, 1 mM EDTA); Hb was subsequently eluted using a gradient from 80% to 85% Buffer Hb2. The purified Hb was dialyzed into storage buffer (10 mM HEPES pH 7.2, 1 mM EDTA), aliquoted, flash-frozen in liquid nitrogen, and stored at −80°C. The concentration and oxidation state of oxyHb were assessed by UV–Vis absorption spectroscopy using known molar extinction coefficients for heme-specific absorbance peaks.^39^

Strep-tagged IsdB was expressed and purified as described previously.^40^ Strep-tag II-IsdB (residues 125–485, UniProt Q8NX66) was codon-optimized for *E*. *coli*, cloned into pASK-IBA3-plus vector, and expressed in *E. coli* BL21 strain induced with 0.2 μg/mL anhydrotetracycline at 20°C for 20 h. Cells were lysed, and the protein purified using Strep-TactinXT affinity resin followed by SEC in Buffer W. Final yield exceeded 100 mg/L with >95% purity. Protein concentration was calculated using ε_280nm_ = 47,790 M^−1^ cm^−1^, with holo-IsdB content estimated to be <5% based on heme absorbance at 405 nm (ε_405nm_ = 90,500 M^−1^ cm^−1^).^40^

#### Hb co-crystallization with C35

Crystals of carboxyhemoglobin (HbCO) grew in the presence of 10 mM C35 in a solution containing 3.0 M ammonium sulfate and 1% (w/v) 2-methyl-2,4-pentanediol (MPD) in sitting drop conditions after which the crystals were cryo-cooled in liquid nitrogen for data collection. X-ray diffraction data were collected at Diamond Light Source beamline i03 at wavelength 0.9763 Å. Data were integrated and scaled using the CCP4 package; structures were solved by molecular replacement using Phaser crystallographic software.^41^ The structural model was iteratively refined and rebuilt by using the Coot program.^42^ Ligand coordinates and restraints were generated from the SMILES string using the Grade software package (Global Phasing Ltd). The structure coordinates are deposited on Protein DataBank (PDB) (PDB: 28OD), and the data collection and refinement statistics are shown in Table S1.

To investigate the presence of global 3D-shape similarity, using the C35-bound Hb as a reference, the ‘‘Structure similarity search’’ tool on the PDB website (https://www.rcsb.org/search/advanced/structure) was exploited. The search was conducted by uploading the C35-bound Hb structure in pdb format without relaxation step and considering only molecular assemblies.

#### Oxygen binding to Hb

Oxygen-binding curves of 100 μM Hb were obtained under ‘stripped’ conditions (*i.e.*, in the absence of any allosteric Hb effectors) using 100 mM HEPES, 1 mM EDTA, pH 7.4 at 37 ± 0.4°C, either with or without the studied effectors. The oxygenated Hb (oxyHb) concentration was estimated using the extinction coefficient at 415 nm of 125,000 M^−1^ cm^−1^. The Hayashi reducing system was exploited to prevent autoxidation^43^ during measurements. Before measurement, oxyHb was deoxygenated under helium (50 mL/min) for 60 min to obtain the deoxygenated form (deoxyHb). Samples were then exposed to different partial oxygen pressures (pO_2_), generated using an Environics 4000 gas mixer (Environics Inc, Tolland, CT, U.S.A.), connected to a helium bottle and different premixed helium/oxygen bottles. Spectra were collected after 30 min incubation time in the 450–700 nm range using a Cary 4000 spectrophotometer (Agilent, Santa Clara, CA, USA). The control experiments, performed in the absence of any ligands, were carried out in the presence of 2% DMSO (v/v).

The Hb fractional saturation at each pO_2_ was calculated by adapting the method reported by Rivetti et al.^44^ Spectra were analyzed as a linear combination of the deoxyHb, oxyHb and metHb spectra (*i.e.*, the reference spectra) measured under the same experimental conditions (Equation 1):

(Equation 1) $$S=S_{0}+a\sum_{i}f_{i}S_{i}$$

Where *S* is the measured absorbance spectrum, *S*_*0*_ is an offset, *a* is the scale factor to correct for the intensity of the analyzed spectrum, the index *i* denotes the heme species (oxyheme, deoxyheme and metheme), *S*_*i*_ are the reference spectra and *f*_*i*_ are the fractional coefficients. The estimation of the oxygen saturation (y) is obtained as (Equation 2):

(Equation 2) $$y=\frac{f_{oxy}}{f_{oxy}+f_{deoxy}}$$

The pO_2_ corresponding to 50% fractional saturation (*p*50) and the Hill coefficient (n) were estimated by fitting the calculated fractional saturation at different pO_2_s to the Hill equation (Equation 3):

(Equation 3) $$y=\frac{{{pO}_{2}}^{n}}{{p_{50}}^{n}+{{pO}_{2}}^{n}}$$

Where *y* is the oxygen saturation, pO_2_ is the partial pressure of oxygen, *p*50 is the oxygen partial pressure corresponding to 0.5 fractional saturation and *n* is the Hill coefficient, accounting for binding cooperativity.

#### Isothermal titration calorimetry

Experiments were performed at 25°C using 12 μM IsdB and 1 mM C35 in a buffered solution containing 50 mM HEPES buffer, pH 7.6. C35 was first dissolved at 100 mM in DMSO and then diluted in the final buffer to a concentration of 1 mM. Solutions were degassed for 10 min under vacuum before the titration. DMSO was added to the IsdB solution at a final concentration of 1% to balance the amount of DMSO in the C35 solution. ITC titrations were carried out using a MicroCal PEAQ-ITC instrument (Malvern, Malvern, UK). C35 was added to the instrument measurement cell, containing 280 μL of IsdB, by a first addition of 0.4 μL and 18 subsequent additions of 2 μL. A time interval of 150 s was set between the addition of each aliquot of C35. To subtract the dilution heat, a reference experiment was performed in which the reaction cell was filled only with the buffer solution with 1% DMSO, while the syringe was filled with 1 mM C35. Experiments were performed under continuous stirring at 750 rpm. All experiments were performed in triplicate. Data analysis was performed using MicroCal PEAQ-ITC Analysis Software (version 1.41, Malvern Panalytical, Malvern, UK).

#### Size-exclusion chromatography

SEC was performed to assess whether compound C35 exerts a destabilizing effect on the Hb:haptoglobin (Hb:Hp) complex and/or on the quaternary structure of isolated Hb (*i.e.*, its tetrameric form).

The destabilizing effect of compound C35 on the Hb:Hp complex was investigated by exploiting an Agilent 1260 HPLC system (Agilent Technologies, Inc., Santa Clara, CA, USA) equipped with a Superdex 200 Increase 3.2/300 column (Cytiva). The column was pre-equilibrated with PBS buffer (pH 7.4) either in the absence or presence of 1 mM C35, and operated at a flow rate of 0.07 mL/min. Samples containing Hb, Hp (Phenotype 1-1) or the Hb:Hp complex (prepared using a 2:1 stoichiometry, *i.e.*, one Hb dimer associated with one Hp protomer), at concentrations of 2 mg/mL, were injected and eluted at 25°C. Elution profiles were monitored by measuring absorbance at 280 nm and 406 nm using an Agilent 1260 Infinity II WR Diode Array Detector.

The estimation of the dissociation constant for the Hb dimer/tetramer equilibrium was carried out using an ÄKTA Pure 25 M chromatographic system (GE Health Sciences, Chicago, IL, USA) equipped with a Superdex 75 Increase 5/150 GL column (GE Health Sciences) with a mobile phase consisting of 20 mM Tris, 150 mM NaCl and 1 mM EDTA pH 8.0 at 20°C, either in the absence or presence of 0.01 mM C35. Control experiments in the absence of C35 were carried out adding 0.01% (v/v) DMSO. The amount of DMSO in the control experiments accounts for the same amount of DMSO related to C35-treated samples. The flow rate was set to 0.3 mL/min. The separation was run at room temperature and the absorbance of the column effluent was monitored both at 280 nm (for calibration) and to 415 nm (for Hb samples). Hb was loaded at the following concentrations: 0.2, 1, 5, 15 and 30 μM. The calibration curve was built using conalbumin (75 kDa), ovalbumin (43 kDa) and lysozyme (14 kDa) as standard proteins (Gel Filtration Calibration Kit HMW, Cytiva). The concentrations reported in Figure S3 also consider column dilution, which was approximately 3-fold with respect to the loading concentrations. Data points in Figure S3 were fitted using (Equation 4):

(Equation 4) $$y=a\frac{2\left[Hb\right]+K_{D}-\sqrt{K_{D}\left(4\left[Hb\right]+K_{D}\right)}}{2\left[Hb\right]}+y_{o}$$

where *y* is the estimated apparent molecular weight, *a* is the amplitude, corresponding to the apparent molecular weight of the tetramer, [Hb] is the Hb concentration, *K*_*D*_ is the dissociation constant for the Hb dimer/tetramer equilibrium and *y*_*0*_ is an offset, corresponding to the apparent molecular weight of the Hb dimer.

### Quantification and statistical analysis

Bacterial growth data are presented as the mean ± standard deviation (SD) of three different cultures. Statistical analysis was performed using Student’s *t* test with GraphPad Prism 8.0 (GraphPad Software, Inc., San Diego, CA, USA).
