# Supplementary material for: Two birds with one stone: An antibiotic hit blocking Staphylococcus aureus heme uptake with serendipitous hemoglobin left-shifting activity
Source: iScience. 2026 Apr 7;29(5):115625. doi: 10.1016/j.isci.2026.115625 (PMC13138187; doi:10.1016/j.isci.2026.115625)
Supplement: Document S1. Figures S1–S3 and Tables S1 and S2 [file mmc1.pdf]

iScience, Volume *29*

Supplemental information

Two birds with one stone: An antibiotic hit blocking *Staphylococcus aureus* heme uptake with serendipitous hemoglobin left-shifting activity

Sarah Hijazi, Francesco Marchesani, Marialaura Marchetti, Valeria Buoli Comani, Paul Brear, Barbara Campanini, Luca Ronda, Serena Faggiano, Eleonora Gianquinto, Somayeh Asgharpour Hassankiade, Barbara Rolando, Francesca Spyrakis, Carlotta Compari, Loretta Lazzarato, Omar De Bei, Emanuela Frangipani, and Stefano Bettati

SUPPLEMENTAL INFORMATION

Two birds with one stone: an antibiotic hit blocking *Staphylococcus aureus* heme uptake with serendipitous hemoglobin left-shifting activity

Sarah Hijazi, Francesco Marchesani, Marialaura Marchetti, Valeria Buoli Comani, Paul Brear, Barbara Campanini, Luca Ronda, Serena Faggiano, Eleonora Gianquinto, Somayeh Asghar Pour Hassan Kiyadeh, Barbara Rolando, Francesca Spyrakis, Carlotta Compari, Loretta Lazzarato, Omar De Bei, Emanuela Frangipani, Stefano Bettati

Figure S1. Characterization of the binding of C35 to IsdB

Raw data for ITC titration of 12 μM IsdB with 1 mM C35 (top panel); binding isotherm of the integrated titration curve (bottom panel). The experiment was carried out at 25 °C in 50 mM HEPES buffer, pH 7.6.

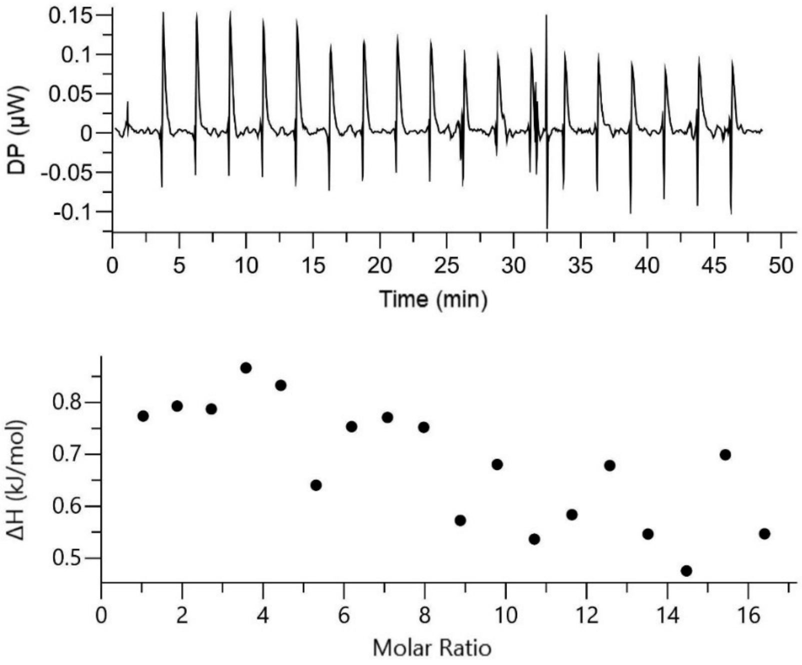

Figure S2. Structural comparison of the crystallographic poses of C35 (PDB ID: 28OD), PF-07059013 (PDB ID: 7JY3) and INN298 (PDB ID: 3IC0) bound to Hb

The protein is shown as cartoons with subunits differently colored and labeled; ligands and binding-site residues are displayed as capped sticks. Hydrogen bonds are shown as black dashed lines. (A) Superposition of Hb bound to C35 (lilac), PF-07059013 (yellow) and INN298 (orange), highlighting the shared interfacial binding region. (B) C35 adopts the most extended binding mode, spanning the interfacial cleft and forming a salt bridge with Arg141, a hydrogen bond with Leu2, and intermolecular π-stacking interactions, suggesting stabilization through both shape complementarity and terminal polar contacts. (C) PF-07059013 binds in the same region but adopts a more compact and bent conformation, with recognition dominated by interactions in the central portion of the cavity, including hydrogen bonds with Leu2 and water-mediated contacts, as well as hydrophobic interactions with Leu2, Pro77 and Tyr140. (D) INN298 extends into subpockets not occupied by the other ligands and, in addition to the covalent bond with Val1, forms hydrophobic contacts with Leu2, Tyr35 and Thr134, consistent with a multi-subpocket binding mode. (E) Superposition of C35 and PF-07059013 illustrating the different degrees of interface spanning and central cavity engagement.

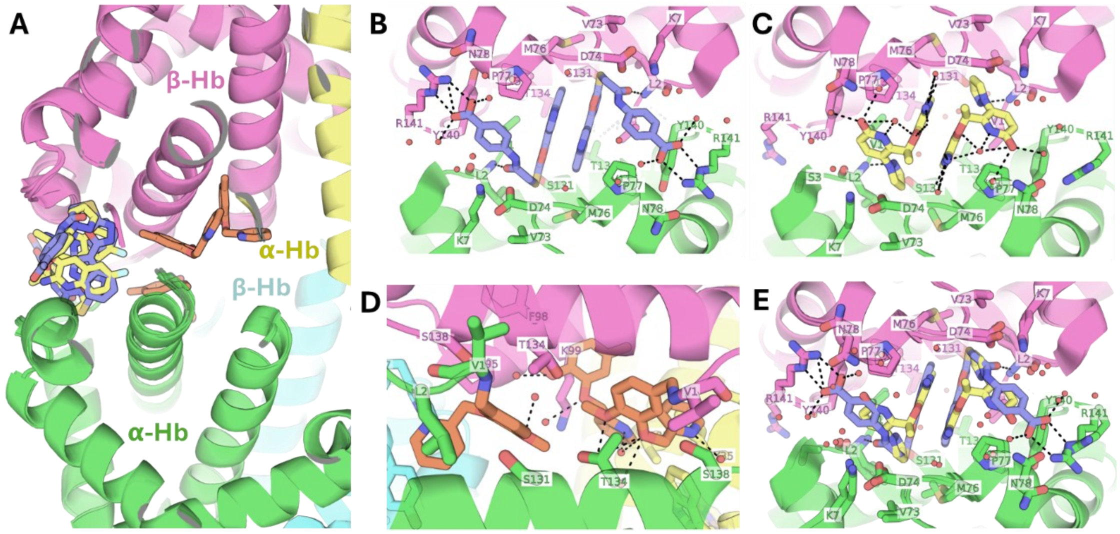

Figure S3. Effect of C35 on the tetramer/dimer equilibrium of oxygenated Hb

(A) Dependence of the apparent molecular weight as a function of Hb concentrations both in the absence (white circles) and presence of 0.01 mM C35 (magenta circles). Data points in the absence of C35 are obtained in the presence of the same DMSO concentration (i.e., 0.1% v/v) with respect to data points obtained in the presence of C35. The fitting of the data (solid lines) to Equation 3 allowed for the estimation of dissociation constants of about 0.67 ± 0.15 and 0.80 ± 0.38 μM for the Hb in the absence and presence of C35, respectively, indicating that the compound does not significantly alter the tetramer-to-dimer equilibrium of Hb. (B) Chromatograms of calibrants (lower panel) used to build the calibration curve (upper panel); CONA, OVA and LISO correspond to conalbumin, ovalbumin and lysozyme, respectively.

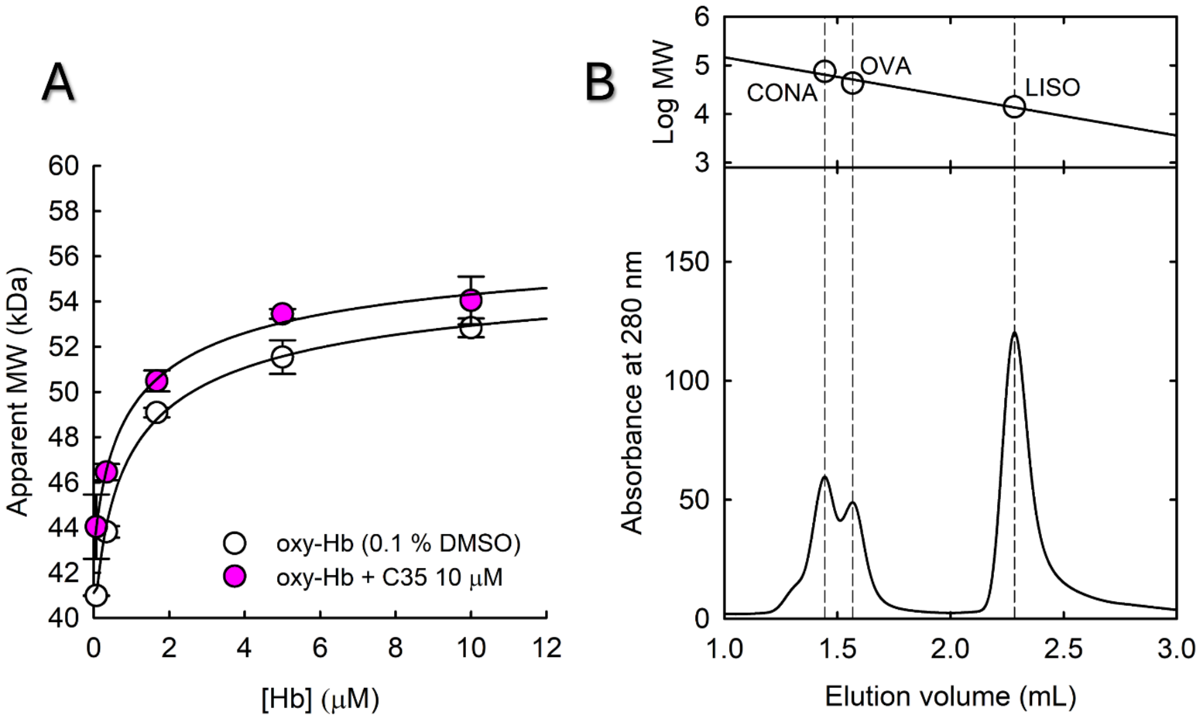

Table S1. Crystal data and structure refinements for Hb:C35 complex

The high-resolution shells are shown in parenthesis.

item	28OD
Collection date	29/01/2023
**Data Collection:**	
Beamline	DIAMOND BEAMLINE I03
Wavelength	0.9763
Resolution range	47.66 - 1.73 (1.760 - 1.730)
Space group	P 32 2 1
Cell (a b c)	92.17 92.17 142.98
Cell (alpha beta gamma)	90.00 90.00 120.00
Total reflections	1474983 (80482)
Unique reflections	73941 (3989)
Multiplicity	19.9 (20.2)
Completeness (%)	100.0 (100.0)
Mean I/sigma(I)	21.1 (2.1)
R-merge	0.079 (1.33)
R-pim	0.018 (0.30)
CC-half	1.000 (0.88)
**Refinement:**	
Refinement resolution range	47.71 - 1.73 (1.77 - 1.73)
No. reflections	70178 (5098)
No. reflections (Rfree)	3697 (324)
R-factor	0.169 (0.239)
Rfree	0.200 (0.275)
Number of total atoms	5172
atoms for macromolecules	4251
atoms for ligands	421
atoms for waters	500
Average B-factor	30.4
RMS(bonds)	0.011
RMS(bond angles)	1.777
RMS(dihedral angles)	5.324
Crystallisation conditions	3.0 M Ammonium sulfate; 1% (w/v) MPD
Ligand SMILES	O=C(CSc1nnc(-c2c[nH]c3ccccc23)o1)Nc1ccc(C(=O)[O-])cc1

Table S2. Hb structures most similar to the Hb:C35 complex retrieved by PDB structure similarity search

PDB ID, score and corresponding DOI of the first ten structures obtained exploiting "Structure similarity search'' tool on the PDB website (https://www.rcsb.org/search/advanced/structure) and using the structure of Hb bound to C35 as a reference.

PDB ID	Rank	Score	Description	Ligand	Organism
6KAS	1	87.22	Carbonmonoxy human hemoglobin A in the R2 quaternary structure at 95 K: Dark	No ligand	Homo sapiens
1QXE	2	87.20	Structural Basis for the Potent Antisickling Effect of a Novel Class of 5-Membered Heterocyclic Aldehydic Compounds	5-hydroxymethyl-furfural	Homo sapiens
6L5X	3	86.82	Carbonmonoxy human hemoglobin A in the R2 quaternary structure at 95 K: Light (2 min)	No ligand	Homo sapiens
3IC0	4	86.50	Crystal Structure of liganded hemoglobin in complex with a potent antisickling agent, INN-298	INN-298	Homo sapiens
6KAT	5	86.18	Carbonmonoxy human hemoglobin A in the R2 quaternary structure at 95 K: Light	No ligand	Homo sapiens
6KAU	6	86.07	Carbonmonoxy human hemoglobin A in the R2 quaternary structure at 140 K: Dark	No ligand	Homo sapiens
6L5Y	7	85.06	Carbonmonoxy human hemoglobin A in the R2 quaternary structure at 140 K: Light (2 min)	No ligand	Homo sapiens
6KAV	8	84.93	Carbonmonoxy human hemoglobin A in the R2 quaternary structure at 140 K: Light	No ligand	Homo sapiens
1QXD	9	84.60	Structural Basis for the Potent Antisickling Effect of a Novel Class of 5-Membered Heterocyclic Aldehydic Compounds	Furfural	Homo sapiens
IC2	10	84.27	Crystal Structure of liganded hemoglobin in complex with a potent antisickling agent, INN-266	INN-266	Homo sapiens
